# Editorial: Regulation of inflammation: metabolic reprogramming and posttranslational modification

**DOI:** 10.3389/fimmu.2026.1823350

**Published:** 2026-03-20

**Authors:** Zhengyu Jiang, Tianliang Li, Jinjun Bian, Wanwan Huai

**Affiliations:** 1 Faculty of Anesthesiology, Changhai Hospital, Naval Medical University, Shanghai, China; 2 National Key Laboratory of Immunity and Inflammation, Naval Medical University, Shanghai, China; 3 Department of Immunology, University of Texas Southwestern Medical Center, Dallas, TX, United States

**Keywords:** ferroptosis, immunometabolism, inflammation, metabolic reprogramming, O-GlcNAcylation, posttranslational modification

Inflammation is regulated by multiple mechanisms that operate at different levels. Among these, metabolic reprogramming and posttranslational modifications have emerged as key factors that shape how immune cells respond to stimuli (1, 2). The observation that activated immune cells undergo metabolic shifts has transformed our understanding of inflammation. This Research Topic brings together five contributions that explore different aspects of this field, from molecular mechanisms to therapeutic applications.

Metabolic reprogramming is now recognized as a hallmark of immune cell activation. When immune cells become activated, they change how they process nutrients to meet the energy and biosynthetic demands of an immune response (3). The hexosamine biosynthetic pathway has emerged as a critical regulator in this process. This pathway produces UDP-GlcNAc, which drives O-GlcNAcylation, a posttranslational modification that affects proteins in the nucleus, cytoplasm, and mitochondria. Through this mechanism, cells can sense nutrient availability and translate it into functional changes.

In this Research Topic, Zhang et al. reports that FTO, an RNA demethylase, undergoes O-GlcNAcylation at serine 95 during LPS stimulation in macrophages. This modification leads to TRIM21-mediated ubiquitination and degradation of FTO, which in turn increases m6A methylation of Socs1 mRNA and sustains SOCS1 protein expression. The result is suppression of inflammatory cytokine production. Their findings reveal a negative feedback mechanism that connects metabolic sensing through the hexosamine biosynthetic pathway to epigenetic regulation of inflammation. This has implications for managing sepsis and other inflammatory conditions.

Another area where metabolism and inflammation intersect is ferroptosis, a form of regulated cell death that depends on iron and lipid peroxidation. Unlike apoptosis or necrosis, ferroptosis has specific metabolic requirements (4). It involves accumulation of polyunsaturated fatty acids, disruption of glutathione metabolism, and altered iron homeostasis. This positions ferroptosis at the crossroads of metabolic reprogramming and inflammatory disease.

Xia et al. review the role of ferroptosis in osteoarthritis. They describe how changes in lipid, amino acid, and iron metabolism create conditions that favor ferroptosis in arthritic joints. Inflammatory cytokines disrupt iron homeostasis in cartilage and synovium. When cells undergo ferroptosis, they release damage-associated molecular patterns and lipid peroxidation products that further activate immune responses. This creates a cycle where inflammation promotes ferroptosis, and ferroptosis amplifies inflammation. The authors discuss therapeutic strategies including iron chelation, antioxidant therapy, and inhibition of lipid peroxidation.

Wang et al. examined how MAGL-18c, an inhibitor of monoacylglycerol lipase, protects against sepsis-associated liver injury. Their study shows that MAGL-18c reduces hepatic inflammation by suppressing TGF-beta signaling and decreasing production of pro-inflammatory cytokines. At the same time, the compound improves liver histology, reduces neutrophil infiltration, and prevents hepatocyte apoptosis and mitochondrial dysfunction. These effects are linked to changes in medium and long-chain fatty acid metabolism. This work demonstrates that modulating lipid metabolism can reduce organ damage in systemic inflammatory conditions.

Systems biology approaches are also advancing the field by identifying biomarkers and therapeutic targets (5). Machine learning and transcriptomic analysis can reveal patterns that are not apparent from studying individual molecules or pathways. These methods are particularly useful for complex conditions where multiple factors contribute to disease.

Wu et al. apply machine learning to identify biomarkers for acute respiratory distress syndrome. They identify SMARCD3 and TCN1 as key markers associated with immune cell function and metabolic reprogramming. An artificial neural network model using these biomarkers shows good predictive performance for ARDS onset. Functional studies confirm that these molecules affect mitochondrial function, oxidative stress, and glucose metabolism. This work illustrates how computational approaches can be combined with experimental validation to advance understanding of inflammatory disease.

Wen et al. review metabolic reprogramming in rheumatoid arthritis, with attention to how traditional Chinese medicine modulates these pathways. They discuss how TCM formulations affect glycolysis, lipid metabolism, mitochondrial function, and glutamine metabolism. By targeting multiple metabolic axes, these interventions can reduce pro-inflammatory responses. This approach represents an alternative strategy for developing therapies that act on metabolism rather than targeting single inflammatory mediators.

Several themes emerge from these contributions. First, the hexosamine biosynthetic pathway and O-GlcNAcylation serve as a link between nutrient availability and immune function. Second, metabolic changes can directly affect cell fate decisions, including ferroptosis. Third, targeting metabolic pathways offers therapeutic potential for inflammatory diseases. Whether through small molecules, natural compounds, or metabolic enzymes, interventions that modulate metabolism can influence inflammatory outcomes.

Challenges remain in translating these findings to clinical practice. Metabolic regulation varies between cell types and depends on context (6). Methods such as single-cell metabolomics and spatial transcriptomics will be needed to understand metabolic heterogeneity in inflammatory microenvironments. Developing targeted delivery systems and validating metabolic biomarkers will also be important for bringing metabolic therapies to patients.

We hope the work presented in this Research Topic will encourage further investigation into how metabolism regulates inflammation and contribute to the development of new therapies for patients with inflammatory diseases.
